# Digital HIV Care Navigation for Young People Living With HIV in San Francisco, California: Feasibility and Acceptability Study

**DOI:** 10.2196/16838

**Published:** 2020-01-10

**Authors:** Dillon Trujillo, Caitlin Turner, Victory Le, Erin C Wilson, Sean Arayasirikul

**Affiliations:** 1 Center for Public Health Research San Francisco Department of Public Health San Francisco, CA United States; 2 Departments of Psychiatry and Pediatrics University of California, San Francisco San Francisco, CA United States

**Keywords:** HIV/AIDS, digital HIV care navigation, young people living with HIV, mHealth

## Abstract

**Background:**

HIV continues to be a public health challenge adversely affecting youth and young adults, as they are the fastest-growing group of new HIV infections in the United States and the group with the poorest health outcomes among those living with HIV. HIV prevention science has turned to mobile health as a novel approach to reach and engage young people living with HIV (YPLWH) experiencing barriers to HIV care.

**Objective:**

This study aimed to assess the feasibility and acceptability of a text message–based HIV care navigation intervention for YPLWH in San Francisco. Health eNavigation is a 6-month text message–based HIV care navigation where YPLWH are connected to their own HIV care navigator through text messaging to improve engagement in HIV primary care. Digital HIV care navigation included delivery of the following through SMS text messaging: (1) HIV care navigation, (2) health promotion and education, (3) motivational interviewing, and (4) social support.

**Methods:**

We evaluated the feasibility and acceptability of a text message–based HIV care navigation intervention among YPLWH. We assessed feasibility using quantitative data for the overall sample (N=120) to describe participant text messaging activity during the intervention. Acceptability was assessed through semistructured, in-depth interviews with a subsample of 16 participants 12 months after enrollment. Interviews were audio-recorded, transcribed, and analyzed using grounded theory.

**Results:**

Overall, the text message–based HIV care navigation intervention was feasible and acceptable. The majority of participants exhibited medium or high levels of engagement (50/120 [41.7%] and 26/120 [21.7%], respectively). Of the majority of participants who were newly diagnosed with HIV, 63% (24/38) had medium to high engagement. Similarly, among those who were not newly diagnosed, 63% (52/82) had medium to high engagement. The majority of participants found that the intervention added value to their lives and improved their engagement in HIV care, medication adherence, and viral suppression.

**Conclusions:**

Text message–based HIV care navigation is a potentially powerful tool that may help bridge the gaps for linkage and retention and improve overall engagement in HIV care for many YPLWH. Our results indicate that participation in text message–based HIV care navigation is both feasible and acceptable across pervasive structural barriers that would otherwise hinder intervention engagement.

## Introduction

### Background

HIV continues to be a public health challenge adversely affecting youth and young adults, as they are the fastest-growing group of new HIV infections in the United States. According to the Centers for Disease Control and Prevention (CDC), people aged 13 to 24 years accounted for 21% of all new HIV diagnoses in 2016, and approximately 81% of those cases were among young men who have sex with men (MSM) [1]. The CDC also states that 2351 transgender people were diagnosed with HIV in the United States from 2009 to 2014, almost all of whom were trans women [2]. In San Francisco, young people living with HIV (YPLWH) aged 13 to 24 years accounted for nearly 14% of the 223 new HIV cases in 2016, with young MSM and trans women accounting for majority of the cases [3]. Furthermore, young trans women are at equal or greater risk of HIV infection as young MSM, with one in five young trans women being infected with HIV before the age of 25 years [4-6].

YPLWH have poorer outcomes across the HIV care continuum. Specifically, only 63% of youth aged 13 to 24 years and 29% of young adults aged 25 to 34 years who are newly diagnosed with HIV achieve viral suppression 12 months after linkage to care in San Francisco [3]. Previous research has shown that structural barriers such as poverty, homelessness, mental health diagnoses, substance use, increased stigma, and the lack of access to personalized HIV care may explain poorer HIV care outcomes among YPLWH [7-12]. These findings emphasize the need for HIV care interventions among YPLWH, particularly among those with intersecting sexual and gender minority identities who disproportionately experience structural barriers to health [13,14]. It is imperative that interventions consider the structural inequities that YPLWH, particularly young MSM and trans women, face and utilize technology to disrupt the current standard of care that traditional HIV clinics provide.

HIV prevention science has turned to mobile health (mHealth) as a novel approach to reach and engage YPLWH experiencing barriers to HIV care. Various studies suggest that utilizing mHealth technologies such as text messaging is acceptable, feasible, and effective for improving outcomes along the HIV care continuum [15-21]. This study describes Health eNavigation (Health eNav), a text message–based HIV care navigation intervention, where YPLWH are connected to their own HIV care navigator through text messaging to improve engagement in HIV primary care. In this study, we assess the feasibility and acceptability of digital HIV care navigation as an mHealth intervention for YPLWH in San Francisco.

### Overview of Health eNavigation

Health eNav is a 6-month text message–based HIV care navigation where YPLWH are connected to their own HIV care navigator through text messaging to improve engagement in HIV primary care. The intervention included delivery of the following: (1) HIV care navigation, (2) health promotion and education, (3) motivational interviewing, and (4) social support. *HIV care navigation* guides participants in knowing where, when, and how to access all health and related services and increases access to appropriate resources (eg, primary medical care, mental health care, housing, insurance, and benefits) [22]. *Health promotion and education* ensures optimal health literacy for all participants by providing information on the biology of HIV, disease management, communication with providers, risk reduction and healthy behavior, and antiretroviral therapy (ART) adherence. Health promotion content is tailored, personalized and specific to the needs of each participant, documented in their individual care plan, and updated on an ongoing basis. Health promotion and education are delivered to meet participants’ education, developmental, language, gender, sexual, and cultural needs. *Motivational interviewing* is a technique and a style of counseling that can help resolve the ambivalence that prevents patients from realizing their personal goals. Motivational interviewing is directive and aims at eliciting self-motivational statements and behavioral change from the client in addition to creating client discrepancy to enhance motivation for positive change [23,24]. Motivational interviewing activates the capability for beneficial change that everyone possesses [25]. *Social support* is provided through establishing an open, nonjudgmental care relationship between participants and their HIV care navigator to address life events and topics most important to YPLWH that may not be solely focused on their HIV care. Active listening, joint problem-solving, and peer counseling were provided on an as-needed and ongoing basis during the 6-month intervention period.

## Methods

### Ethics Approval

All procedures performed in studies involving human participants were in accordance with the ethical standards of the institutional and national research committee and with the 1964 Helsinki declaration and its later amendments or comparable ethical standards. This study was approved by the Institutional Review Board at the University of California, San Francisco (IRB #16-19675).

### Eligibility and Participant Recruitment

Eligible participants were youth and young adults, aged 18 to 34 years, diagnosed with HIV infection who identified as a man who has sex with men or a trans woman, and reported living in San Francisco. Eligible participants also met at least one of the following criteria: (1) newly diagnosed with HIV (people who have tested HIV positive for the first time within the last 12 months before enrollment), (2) not linked to HIV medical care (people who are aware of their HIV infection status but have never engaged in care or never had an HIV medical visit after being diagnosed with HIV), (3) out of care (people diagnosed with HIV more than 12 months before enrollment who had a gap in their HIV care that was >6 months within the last 24 months), and (4) not virally suppressed (people who have a viral load of ≤200 copies/mL at their last laboratory test). If participants did not have access to a mobile phone, they were provided with a mobile phone and 2 years of cellular service with unlimited text messaging.

Recruitment of participants occurred between January and December 2017. Nonprobability, convenience, and venue-based sampling were used to recruit potential participants from San Francisco Department of Public Health clinics, AIDS service organizations, community-based organizations, and lesbian, gay, bisexual, transgender, and queer youth service providers. Recruiting materials (eg, flyers) and presentations to staff were used to advertise study recruitment. Staff referred potential participants to the study through phone and email communication and/or in-person meetings. Enrolled participants were also invited to refer peers from their social network. We screened 171 potential participants and 140 were eligible, of which 20 individuals were lost to follow-up following the screening, and 120 participants were enrolled into the study.

### Study Procedures and Data Collection

Once screened, eligible participants were educated about the study and provided informed consent and completed administrative paperwork. Participants then met with their HIV care navigator in person and completed a short qualitative interview, a comprehensive care plan, and a computer-assisted self-interviewing (CASI) surveys. Participants and their HIV care navigator developed a comprehensive care plan together that identified at least three unmet needs and intentions for health-related behavior change. The comprehensive care plan identified specific responsibilities and follow-up actions for both the HIV care navigator and participants to work on jointly during the intervention period. This helped to tailor and personalize HIV care navigation to each participant. After 3 months, the HIV care navigator scheduled an in-person informal follow-up appointment to check-in and update comprehensive care plans and keep participants engaged and retained in the study activities.

During the 6-month intervention period, participants were able to communicate with their HIV care navigator via text messaging on an open schedule, and conversations spanned any topic that the participants wanted to discuss. The HIV care navigator sent weekly check-in messages to participants that included the following topics: general well-being, health education and health promotion, social support, and primary care appointment reminders. CASIs and electronic medical chart abstraction were administered in person every 6 months for 18 months. Data collection included a variety of socio-behavioral and HIV care continuum constructs, including information on substance use, mental health, engagement in HIV care, HIV stigma, and social media technology use. Each participant was eligible to receive US $590 in gift cards at baseline for completing all study-related activities over the duration of 18-month follow-up period.

### Data Analysis

#### Demographics

At enrollment, participants were asked to provide their date of birth, and their age was calculated in real time. Participants were asked to self-identify their race and ethnicity. Gender identity was assessed with a 2-question measure, first asking participants to identify their sex assigned at birth and then their current gender identity. Housing status was measured by asking participants to identify which of the following best describes their current housing status: lives with family member, friend or partner who rents or owns a home, temporary or transitional housing, homeless or living in a shelter, or rent or own an apartment or house. Income was assessed by asking participants to self-report their income in the previous month from the following response categories: US $0 to US $250, US $251 to US $600, US $601 to US $1300, or US $1301 or more. Education was measured by self-report of the highest level of education participants received. Incarceration was measured dichotomously by asking if participants were incarcerated in the last 6 months (yes or no). Competing needs was measured dichotomously (yes or no) by asking participants the following two questions: “In the past 6 months, have you ever gone without HIV medications because you needed money for food, clothing, housing, or other basic needs?” and “In the past 6 months, have you ever gone without food, clothing, housing, or other basic needs because you needed the money for HIV medications?”. HIV diagnosis status was measured by asking participants if they were diagnosed with HIV in the last year (newly diagnosed) or if they were diagnosed with HIV more than 1 year ago (not newly diagnosed).

#### Intervention Feasibility

We assessed feasibility using quantitative data for the overall sample (N=120) to describe participant engagement with the intervention. We collected back-end data from a third-party text messaging platform. All text messages sent during the 6-month intervention period between an individual participant and their HIV care navigator were captured and maintained locally in a database and included the date, time, and body of each text message sent. We include quantitative feasibility data for the overall sample (N=120) and, in particular, for HIV diagnosis status to examine whether a text message–based HIV care navigation intervention would be feasible for either (or both) groups of people diagnosed with HIV within the last year and those who were not.

#### Intervention Acceptability

We assessed acceptability by conducting semistructured, in-depth interviews with a subsample of 16 participants 12 months after enrollment. Participants were purposively sampled to obtain diversity in levels of engagement, race or ethnicity, and gender identity. Participants were provided with US $75 in gift cards for their time. Interviews lasted 30 to 45 min and took place during a time that was most convenient for the participant. The interview guide was iterated to maximize coverage of participant experiences through theoretical sampling to reach theoretical saturation and to address the following research question, “What factors impacted acceptability of digital HIV care navigation for young people living with HIV?” [26]. The interview guide assessed the following constructs: overall acceptability, length, and individual and health-related impacts. Interviews were audio-recorded and transcribed verbatim. Transcriptions were randomly checked for quality and accuracy against original recordings. Qualitative interview data were coded and analyzed using grounded theory [26]. Two members of the research team independently coded qualitative data, line by line, and together organized codes into categories to identify specific factors that shaped the acceptability of digital HIV care navigation for YPLWH.

## Results

### Study Sample Demographic Characteristics

Table 1 shows that the majority of the participants identified as male (103/120, 85.8%), and about 14.2% (17/120) of the participants identified as trans women. The mean age of participants was 27 years, and the majority of participants completed high school/General Equivalency Diploma or some college and/or higher level of education (107/120, 89.2%). Overall, our sample was diverse, with nearly three-quarters of participants who identified as Hispanic or Latinx, black, and other or multiple race/ethnic identities (38/120, 31.7%; 22/120, 18.3%; and 28/120, 23.3%, respectively). More than two-thirds of the participants were diagnosed with HIV more than 1 year ago (82/120, 68.3%), and approximately one-third of the participants were diagnosed with HIV in the last year (38/120, 31.7%). Half of the participants reported being homelessness or living in temporary or transitional housing (60/120, 50.0%).

### Feasibility

On the basis of the number of text messages sent between each participant and their digital HIV care navigator, we defined a low level of engagement as less than 50 or approximately 8 text messages a month during the 6-month intervention period, medium as 50 to 149 or approximately 9 to 24 text messages a month, and high as >150 or 25 or more text messages a month. The number of text messages per participant ranged from 1 to 467 over the intervention period. Overall, the majority of participants exhibited medium or high levels of engagement (50/120, 41.7% and 26/120, 21.7%, respectively). Of the majority of participants who were newly diagnosed with HIV, 63% (24/38) had medium to high engagement. Similarly, among those who were not newly diagnosed, 63% (52/82) had medium to high engagement.

### Acceptability

Overall, 88% (14/16) of participants found that digital HIV care navigation was acceptable. In particular, participants expressed that digital HIV care navigation was motivating and working with their personal digital HIV care navigator was novel and integral to their lives. A participant (white, 25 years old) said:

It was nice to have that check-up. Even my case managers don’t do that a lot of the time. It was just helpful, it’s nice to have another resource available. It felt nice to receive the support through text message, it proved that community sense, it’s like having a friend there that cares for your care, it felt good.

The intervention bridged important gaps to support YPLWH in making and sustaining the changes necessary to engage in HIV care. A participant (white, 28 years old) said:

Now I take my meds every day and I see my doctor, and if I miss my doctor appointment, I immediately go in to reschedule it. When they say “Health eNav,” it really helped you navigate your health and I really liked that. It has definitely been a positive experience. It really helped that [the digital HIV care navigator] was actually there, I was able to text and call [them], [they] were able to go with me to my appointments, and Health eNav got me to a point in my life where my health is my number one priority. I don’t know where I would be without this.

Most people (13/16, 81%) found the substantive content provided through digital HIV care navigation as the main reason for its acceptability. They felt encouraged by the interactions with their digital HIV care navigator. A participant (Hispanic/Latinx, 34 years old) said:

One of the times I went to the doctor and we [participant & digital navigator] were corresponding the entire time. I was in the waiting room and actually in the doctor’s office texting the digital navigator and discussing the questions that I was going to ask the doctor and some of the information that I wanted to make sure to tell her. I needed the digital navigator to help me remember the questions that were important for me to ask which was good because I had always been told to write things down before. That was the first time I actually went to my doctor’s appointment prepared.

### Intervention Length

According to the participants, most (11/16, 69%) felt that 6 months of digital HIV care navigation was a good amount of time for them to acquire new knowledge and information and engage with their HIV care. A participant (white, 24 years old) said:

I think six months is a good amount of time to actually get to know someone enough and to actually figure out their full medical care and figure out where they're at. You also get to see how the person's life has changed over that time and six months doesn’t sound like a lot of time for someone who has all their stuff together but for someone like me who's still in the process of figuring out this and that, a lot of things can change in that time.

### Individual- and Health-Related Impacts

All (16/16, 100%) participants expressed that digital HIV care navigation impacted them in a positive way and improved their engagement in HIV care. A participant (white, 27 years old) said:

It helped me to remember that things are okay, the stigma is still there, but as long as you live healthy and do what you need to do, you’re fine! I also gained information that I would have never though through digital HIV care navigation, like new meds that they’re coming out with, the new tests that they’re trying to do, and the things that they’re trying to do to help us live longer and healthier lives. I would have never of done that sort of research by myself. This access was beneficial.

**Table 1:** Sample characteristics, overall and by engagement level over the 6-month study period, Health eNavigation, 2017-2018 (N=120).

Construct			Overall^a^	Low: <50 texts^b^	Medium: 50-149 texts^b^	High: 150 texts or more^b^
Total, n (%)			120 (100.0)	44 (36.7)	50 (41.7)	26 (21.7)
**Demographics**						
	Age, mean (SD)		27.75 (4.07)	27.05 (4.13)	28.38 (4.31)	27.73 (3.37)
	**Race or ethnicity, n (%)**					
		Black or African American	22 (18.3)	7 (31.8)	8 (36.4)	7 (31.8)
		Hispanic or Latinx	38 (31.7)	18 (47.4)	12 (31.6)	8 (21.1)
		Other^c^ or multiple	28 (23.3)	9 (32.1)	11 (39.3)	8 (28.6)
		White	32 (26.7)	10 (31.3)	19 (59.4)	3 (9.4)
	**Gender identity, n (%)**					
		Cisgender man	103 (85.8)	39 (37.9)	42 (40.8)	22 (21.4)
		Trans woman	17 (14.2)	5 (29.4)	8 (47.1)	4 (23.5)
**Socioeconomic factors**						
	**Housing status, n (%)**					
		Lives with a family member, friend, or partner who rents or owns a home	21 (17.5)	6 (28.6)	7 (33.3)	8 (38.1)
		Temporary or transitional housing^d^	43 (35.8)	16 (37.2)	22 (51.2)	5 (11.6)
		Homeless or shelter	17 (14.2)	9 (52.9)	4 (23.5)	4 (23.5)
		Rents or owns an apartment or house	39 (32.5)	13 (33.3)	17 (43.6)	9 (23.1)
	**Income in the previous month (US $), n (%)**					
		601-1300	30 (25.0)	11 (36.7)	12 (40.0)	7 (23.3)
		251-600	30 (25.0)	13 (43.3)	12 (40.0)	5 (16.7)
		0-250	30 (25.0)	11 (36.7)	13 (43.3)	6 (20.0)
		≥1301	29 (24.2)	9 (31.0)	12 (41.4)	8 (27.6)
	**Education, n (%)**					
		High school or General Equivalency Diploma	39 (32.5)	17 (43.6)	15 (38.5)	7 (18.0)
		Less than high school	13 (10.8)	5 (38.5)	6 (46.2)	2 (15.4)
		Some college or more	68 (56.7)	22 (32.4)	29 (42.7)	17 (25.0)
	**Incarceration in the last 6 months,** **n (%)**					
		Yes	23 (19.2)	13 (56.5)	7 (30.4)	3 (13.0)
		No	97 (80.8)	31 (32.0)	43 (44.3)	23 (23.7)
	**Competing needs, n (%)**					
		Went without HIV medications because needed money for basic needs (eg, food, housing, and clothing)	38 (31.7)	18 (47.4)	13 (34.2)	7 (18.4)
		Went without basic needs (eg, food, housing, and clothing) to have money for HIV medications	32 (26.7)	18 (56.3)	10 (31.3)	4 (12.5)
**HIV diagnosis status, n (%)**						
	Diagnosed in the last year		38 (31.7)	14 (36.8)	11 (29.0)	13 (34.2)
	Diagnosed more than 1 year ago		82 (68.3)	30 (36.6)	39 (47.6)	13 (15.9)

^a^Percentages calculated out of the total number of participants in Health eNav (n=120), unless otherwise specified.

^b^Percentages row calculated out of the total number of participants in each demographic, structural barrier, or HIV diagnosis category.

^c^“Other” race or ethnicity included participants who identified as American Indian or Alaska Native (n=6) or Asian (n=7).

^d^“Temporary or transitional housing” included participants who lived in single-room occupancy hotels, motels, boarding houses, halfway houses, drug treatment centers, independent living units, domestic violence shelters, battered persons’ shelters, or “safe houses.”

### Potential Challenges and Barriers

Three participants who felt that the length of the digital HIV care navigation was too short discussed how experiencing complex barriers to HIV care, which were primarily structural and temporal with regard to their HIV diagnosis status, detracted from their ability to focus on their participation in the intervention; as a result, they would have preferred to have a longer intervention period. A participant (black, 24 years old) said:

If it [the participant’s enrollment in the intervention] were at a different time of me knowing my status, since it was so soon after I was diagnosed, I kind of didn’t want to do or talk about anything related to HIV.

For one participant, their active substance use prevented them from being able to prioritize their HIV care engagement and participation in the intervention. This participant (native American, 33 years old) said:

One thing that was impacting it [their participation in the intervention] was my [substance] use. ‘Cause I would disappear on weeks and months at a time and no one would know where I was, then one day I would just show up. One time I was all the way in Bakersfield, but I made it back to San Francisco...I would disappear, it’s just my little devil on my shoulder pulling me one way, the angel never wins.

## Discussion

### Principal Findings

The Health eNav intervention for young MSM and trans women living with HIV was both feasible and acceptable. Participants unanimously agreed that participating in the intervention impacted them positively and improved their engagement in HIV care. Notably, this paper assessed the potential influence of structural factors and HIV diagnosis (within the last year or longer) as barriers to the intervention. These are vital components to examine, particularly among YPLWH and those with sexual and gender minority identities.

Although several mHealth interventions in the literature have demonstrated that text messages improve HIV outcomes, YPLWH are still experiencing poor outcomes when it comes to linkage and retention to HIV care as well as achieving viral suppression [19]. Much of the new research examining mHealth interventions for engagement in HIV care has solely focused on mHealth to support ART adherence; although these contributions are important, research is overlooking how to implement mHealth interventions within disadvantaged communities, such as young MSM or young trans women living with HIV [17], who disproportionately experience homelessness, incarceration, poverty, and other structural barriers that may hinder participation in HIV interventions [27,28].

Findings from Health eNav provide preliminary evidence of study feasibility across structural barriers previously shown to hinder intervention engagement [27-29]. Overall, a majority of participants (>50%) were classified as having medium or high levels in texting across gender identity, income, and education level. There were some barriers that persisted among this study sample. Notably, participants who were homeless or living in a shelter, those who were recently incarcerated, and those who went without basic needs to afford HIV medications were more likely to be low text engagers. These findings add evidence that basic needs and HIV care are in conflict for marginalized populations [30-32]. Still, almost half of those who were homeless, incarcerated, or experiencing competing needs managed to have medium and high levels of engagement in Health eNav. It could be that mHealth interventions such as Health eNav, which utilize a digital HIV care navigation system that provides personalized instrumental, social, and emotional support, have the capacity to reach across sociodemographic groups and effectively improve HIV care engagement; further research is needed to more definitively confirm this. Interestingly, most of the young MSM and trans women in Health eNav who found the intervention length to be too short explained that structural barriers (eg, homelessness and poverty) contributed to lower participation. Had the intervention stretched over a longer period, these participants felt that they could have taken the time needed to address these barriers and focus on the intervention.

Regardless of HIV diagnosis status (within the last year or earlier), participants in the study tended to be moderate or high engagers in the intervention. However, the acceptability responses of one participant noted that discussing HIV-related topics so soon after diagnosis was difficult. This same participant (black, 24 years old) went on to suggest the following:

It would have been better if I had the option for 3-6-9-12 months.

Future digital HIV care navigation interventions should consider whether adaptable intervention exposure periods or stepped study designs would better meet the needs of YPLWH.

### Limitations and Future Research

These findings should be interpreted with some limitations in mind. First, the study sample here represents young MSM and trans women living with HIV and who have a connection—albeit, possibly, a poor connection—with HIV care or community organizations. In addition, qualitative acceptability data were gathered for a small number of participants. Despite the subsequently limited generalizability of results, the Health eNav intervention enrolled a diverse segment of the target population (young MSM and trans women living with HIV) that represents a multitude of sociodemographic experiences. The study findings may also be subject to measurement bias, in that the categorizations of texting engagement may lose nuance in characterizing the true underlying pattern of intervention engagement. Future research on how to meaningfully characterize and analyze text messaging patterns and mHealth intervention–related big data is needed.

### Conclusions

Regardless of the study limitations, digital HIV care navigation is a potentially powerful tool that may help bridge the gaps for linkage and retention and improve overall engagement in HIV care for many young MSM and young trans women living with HIV. By utilizing digital technology, Health eNav capitalizes on the familiarity and accessibility of mobile devices and social media platforms to engage with hard-to-reach YPLWH who confront unique barriers to HIV care. Our results indicate that participation in digital HIV care navigation is both feasible and acceptable across pervasive structural barriers that would otherwise hinder intervention engagement.

## Abbreviations

ART: antiretroviral therapy
CASI: computer-assisted self-interviewing
CDC: Centers for Disease Control and Prevention
Health eNav: Health eNavigation
mHealth: mobile health
MSM: men who have sex with men
YPLWH: young people living with HIV
